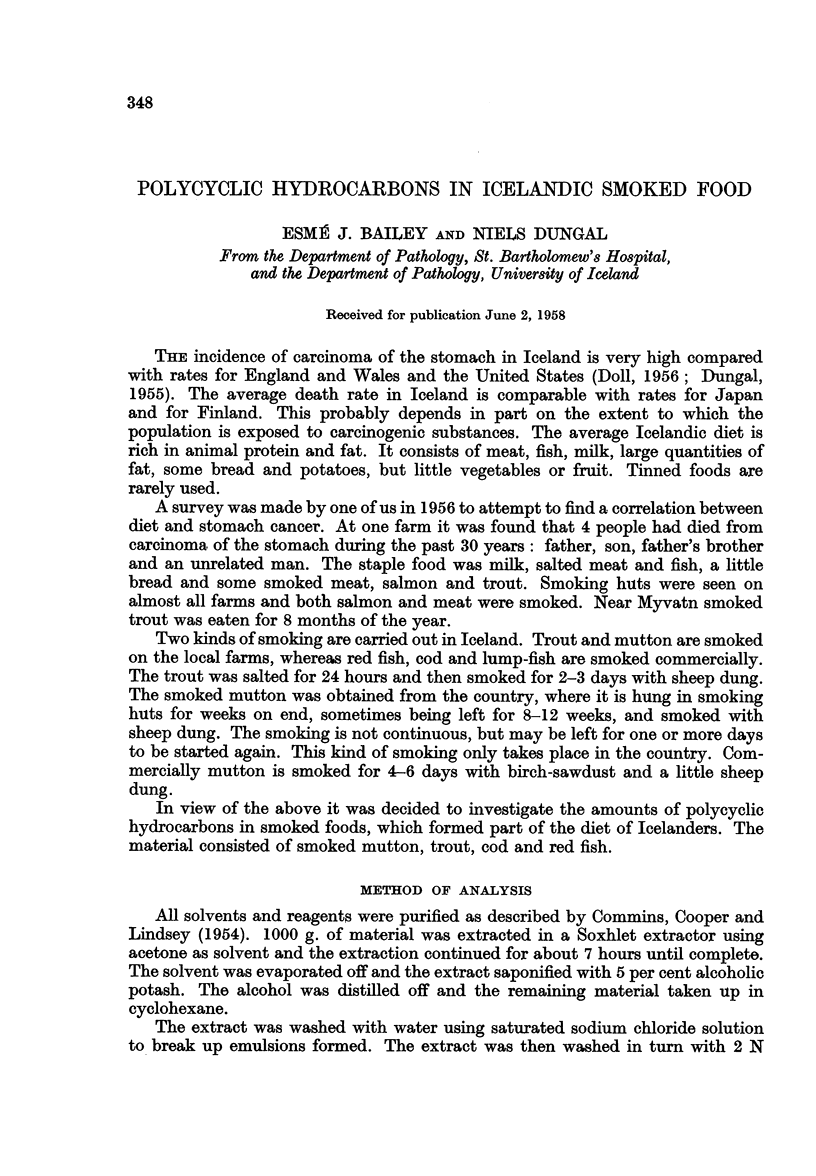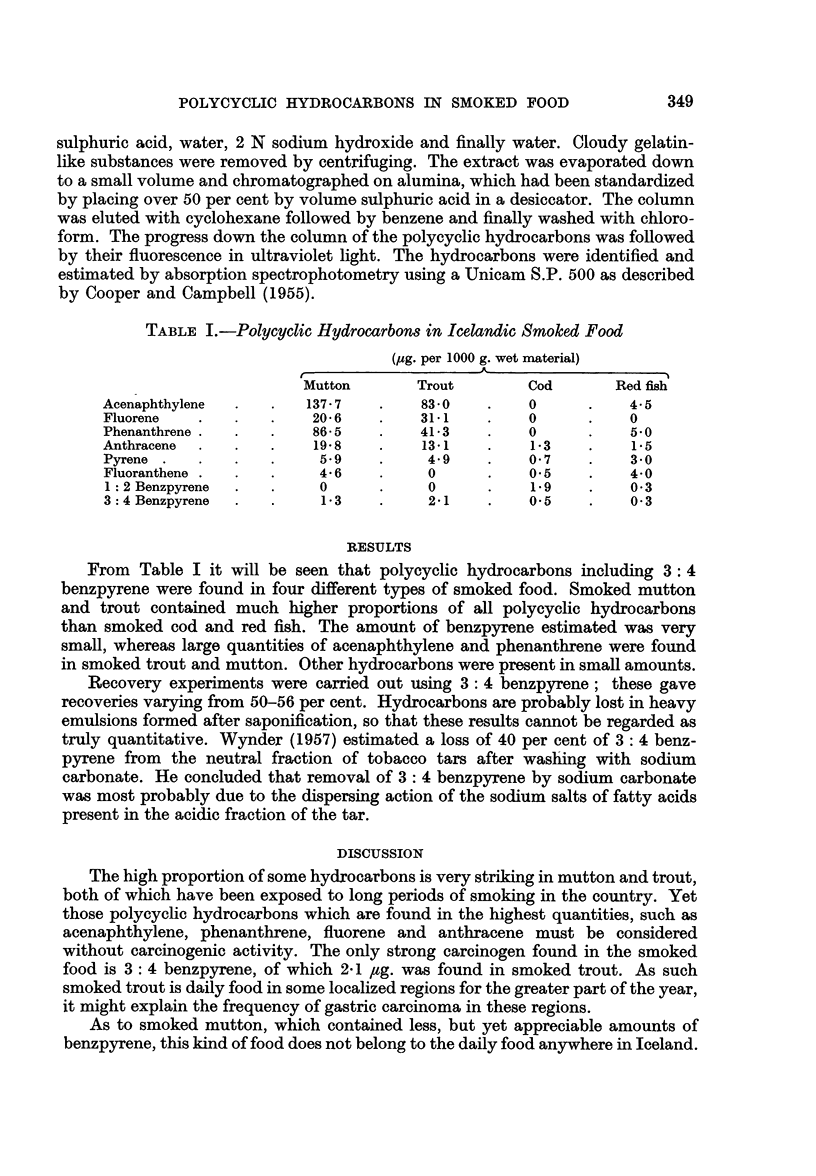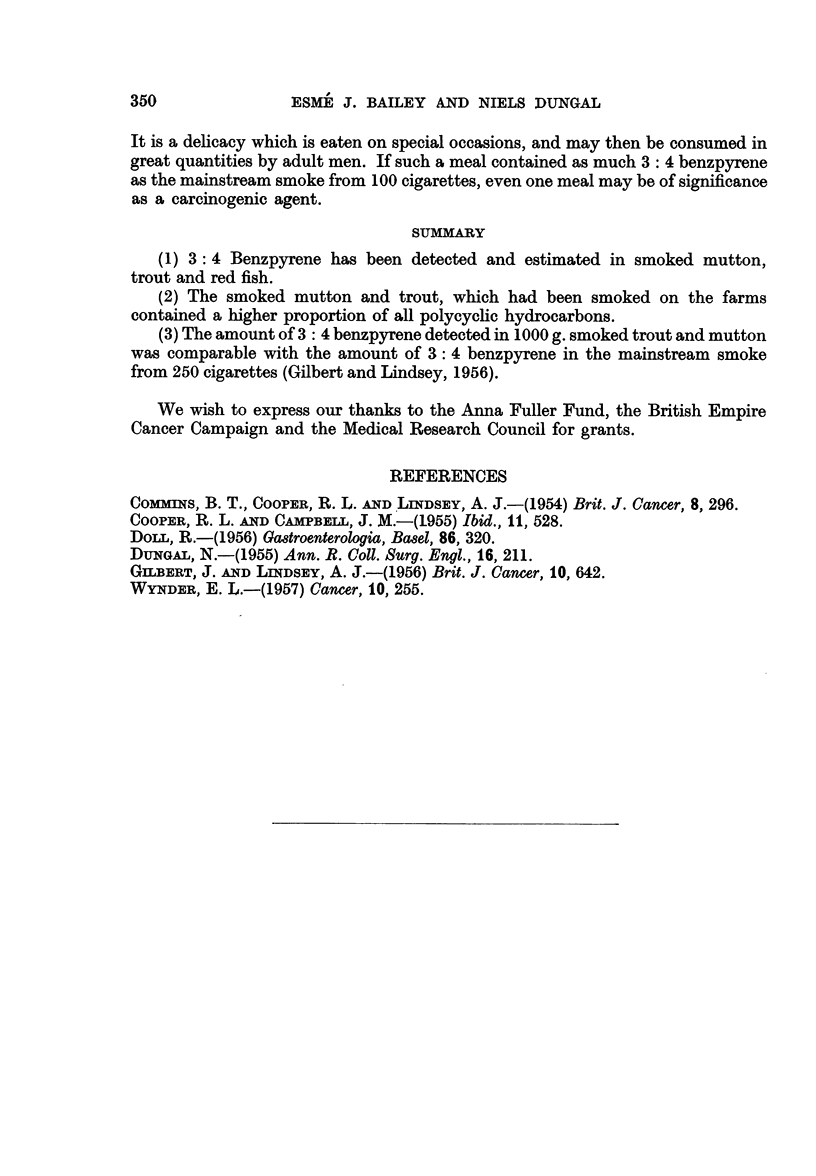# Polycyclic Hydrocarbons in Icelandic Smoked Food

**DOI:** 10.1038/bjc.1958.42

**Published:** 1958-09

**Authors:** Esmé J. Bailey, Niels Dungal


### POLYCYCLIC HYDROCARBONS IN ICELANDIC SMOKED FOOD

ESMÉ J. BAILEY AND NIELS DUNGAL

*From the Department of Pathology, St. Bartholomew's Hospital, and the Department of Pathology, University of Iceland*

Received for publication June 2, 1958

THE incidence of carcinoma of the stomach in Iceland is very high compared with rates for England and Wales and the United States (Doll, 1956 ; Dungal, 1955). The average death rate in Iceland is comparable with rates for Japan and for Finland. This probably depends in part on the extent to which the population is exposed to carcinogenic substances. The average Icelandic diet is rich in animal protein and fat. It consists of meat, fish, milk, large quantities of fat, some bread and potatoes, but little vegetables or fruit. Tinned foods are rarely used.

A survey was made by one of us in 1956 to attempt to find a correlation between diet and stomach cancer. At one farm it was found that 4 people had died from carcinoma of the stomach during the past 30 years : father, son, father's brother and an unrelated man. The staple food was milk, salted meat and fish, a little bread and some smoked meat, salmon and trout. Smoking huts were seen on almost all farms and both salmon and meat were smoked. Near Myvatn smoked trout was eaten for 8 months of the year.

Two kinds of smoking are carried out in Iceland. Trout and mutton are smoked on the local farms, whereas red fish, cod and lump-fish are smoked commercially. The trout was salted for 24 hours and then smoked for 2–3 days with sheep dung. The smoked mutton was obtained from the country, where it is hung in smoking huts for weeks on end, sometimes being left for 8–12 weeks, and smoked with sheep dung. The smoking is not continuous, but may be left for one or more days to be started again. This kind of smoking only takes place in the country. Commercially mutton is smoked for 4–6 days with birch-sawdust and a little sheep dung.

In view of the above it was decided to investigate the amounts of polycyclic hydrocarbons in smoked foods, which formed part of the diet of Icelanders. The material consisted of smoked mutton, trout, cod and red fish.

### METHOD OF ANALYSIS

All solvents and reagents were purified as described by Commins, Cooper and Lindsey (1954). 1000 g. of material was extracted in a Soxhlet extractor using acetone as solvent and the extraction continued for about 7 hours until complete. The solvent was evaporated off and the extract saponified with 5 per cent alcoholic potash. The alcohol was distilled off and the remaining material taken up in cyclohexane.

The extract was washed with water using saturated sodium chloride solution to break up emulsions formed. The extract was then washed in turn with 2 N

sulphuric acid, water, 2 N sodium hydroxide and finally water. Cloudy gelatin-like substances were removed by centrifuging. The extract was evaporated down to a small volume and chromatographed on alumina, which had been standardized by placing over 50 per cent by volume sulphuric acid in a desiccator. The column was eluted with cyclohexane followed by benzene and finally washed with chloroform. The progress down the column of the polycyclic hydrocarbons was followed by their fluorescence in ultraviolet light. The hydrocarbons were identified and estimated by absorption spectrophotometry using a Unicam S.P. 500 as described by Cooper and Campbell (1955).

TABLE I.—*Polycyclic Hydrocarbons in Icelandic Smoked Food*

| | ($\mu$g. per 1000 g. wet material) | | | |
|---|---|---|---|---|
| | Mutton | Trout | Cod | Red fish |
| Acenaphthylene | 137·7 | 83·0 | 0 | 4·5 |
| Fluorene | 20·6 | 31·1 | 0 | 0 |
| Phenanthrene | 86·5 | 41·3 | 0 | 5·0 |
| Anthracene | 19·8 | 13·1 | 1·3 | 1·5 |
| Pyrene | 5·9 | 4·9 | 0·7 | 3·0 |
| Fluoranthene | 4·6 | 0 | 0·5 | 4·0 |
| 1 : 2 Benzpyrene | 0 | 0 | 1·9 | 0·3 |
| 3 : 4 Benzpyrene | 1·3 | 2·1 | 0·5 | 0·3 |

### RESULTS

From Table I it will be seen that polycyclic hydrocarbons including 3 : 4 benzpyrene were found in four different types of smoked food. Smoked mutton and trout contained much higher proportions of all polycyclic hydrocarbons than smoked cod and red fish. The amount of benzpyrene estimated was very small, whereas large quantities of acenaphthylene and phenanthrene were found in smoked trout and mutton. Other hydrocarbons were present in small amounts.

Recovery experiments were carried out using 3 : 4 benzpyrene ; these gave recoveries varying from 50–56 per cent. Hydrocarbons are probably lost in heavy emulsions formed after saponification, so that these results cannot be regarded as truly quantitative. Wynder (1957) estimated a loss of 40 per cent of 3 : 4 benzpyrene from the neutral fraction of tobacco tars after washing with sodium carbonate. He concluded that removal of 3 : 4 benzpyrene by sodium carbonate was most probably due to the dispersing action of the sodium salts of fatty acids present in the acidic fraction of the tar.

### DISCUSSION

The high proportion of some hydrocarbons is very striking in mutton and trout, both of which have been exposed to long periods of smoking in the country. Yet those polycyclic hydrocarbons which are found in the highest quantities, such as acenaphthylene, phenanthrene, fluorene and anthracene must be considered without carcinogenic activity. The only strong carcinogen found in the smoked food is 3 : 4 benzpyrene, of which 2·1 $\mu$g. was found in smoked trout. As such smoked trout is daily food in some localized regions for the greater part of the year, it might explain the frequency of gastric carcinoma in these regions.

As to smoked mutton, which contained less, but yet appreciable amounts of benzpyrene, this kind of food does not belong to the daily food anywhere in Iceland.

It is a delicacy which is eaten on special occasions, and may then be consumed in great quantities by adult men. If such a meal contained as much 3 : 4 benzpyrene as the mainstream smoke from 100 cigarettes, even one meal may be of significance as a carcinogenic agent.

### SUMMARY

(1) 3 : 4 Benzpyrene has been detected and estimated in smoked mutton, trout and red fish.

(2) The smoked mutton and trout, which had been smoked on the farms contained a higher proportion of all polycyclic hydrocarbons.

(3) The amount of 3 : 4 benzpyrene detected in 1000 g. smoked trout and mutton was comparable with the amount of 3 : 4 benzpyrene in the mainstream smoke from 250 cigarettes (Gilbert and Lindsey, 1956).

We wish to express our thanks to the Anna Fuller Fund, the British Empire Cancer Campaign and the Medical Research Council for grants.

### REFERENCES

COMMINS, B. T., COOPER, R. L. AND LINDSEY, A. J.—(1954) *Brit. J. Cancer*, **8**, 296.
COOPER, R. L. AND CAMPBELL, J. M.—(1955) *Ibid.*, **11**, 528.
DOLL, R.—(1956) *Gastroenterologia, Basel*, **86**, 320.
DUNGAL, N.—(1955) *Ann. R. Coll. Surg. Engl.*, **16**, 211.
GILBERT, J. AND LINDSEY, A. J.—(1956) *Brit. J. Cancer*, **10**, 642.
WYNDER, E. L.—(1957) *Cancer*, **10**, 255.